# Colorectal Adenoma Subtypes Exhibit Signature Molecular Profiles: Unique Insights into the Microenvironment of Advanced Precancerous Lesions for Early Detection Applications

**DOI:** 10.3390/cancers17040654

**Published:** 2025-02-14

**Authors:** Francesco Mattia Mancuso, Juan Carlos Higareda-Almaraz, Pol Canal-Noguer, Arianna Bertossi, Alexandre Perera-Lluna, Michael Herbert Alexander Roehrl, Kristi Kruusmaa

**Affiliations:** 1 Universal Diagnostics S.A., 41013 Seville, Spain; francesco.mancuso@universaldx.com (F.M.M.); jc.higareda@universaldx.com (J.C.H.-A.); pol.canal@universaldx.com (P.C.-N.); 2 Research & Development, Universal Diagnostics d.o.o., 1000 Ljubljana, Slovenia; arianna.bertossi@universaldx.com; 3 B2SLab, Institute for Research and Innovation in Health (IRIS), Universitat Politècnica de Catalunya—BarcelonaTech, 08028 Barcelona, Spain; alexandre.perera@upc.edu; 4 Networking Biomedical Research Centre in the Subject Area of Bioengineering, Biomaterials and Nanomedicine (CIBER-BBN), 28029 Madrid, Spain; 5 Department of Pathology, Beth Israel Deaconess Medical Center, Harvard Medical School, Boston, MA 02215, USA; michael_roehrl@bidmc.harvard.edu

**Keywords:** colorectal cancer, adenoma, advanced precancerous lesion, methylation, copy-number alterations, pathway enrichment

## Abstract

**Background**: Colorectal cancer (CRC) is characterized by the uncontrolled growth of malignant colonic or rectal crypt epithelium. About 85% of CRCs evolve through a stepwise progression from advanced precancerous adenoma lesions. A better understanding of the evolution from adenoma to carcinoma can provide a window of opportunity not only for early detection and therapeutic intervention but potentially also for cancer prevention strategies. **Methods**: This study investigates the heterogeneous methylation, copy-number alteration (CNA), and mutation signals of histological adenoma subtypes in the context of progression from normal colon to advanced precancerous lesions (APLs) and early-stage CRC. **Results**: Differential methylation analysis revealed 2321 significantly altered regions among APLs: 137 hypermethylated regions in serrated vs. tubular, 2093 in serrated vs. tubulovillous, and 91 in tubular vs. tubulovillous adenoma subtypes. The most differentiating pathways for serrated adenomas belonged to cAMP signaling and the regulation of pluripotency of stem cells, while regions separating tubular and tubulovillous subtypes were enriched for WNT signaling. CNA events were mostly present in tubular or tubulovillous adenomas, with the most frequent signals being seen in chromosomes 7, 12, 19, and 20. In contrast, early-stage CRC exhibited signals in chromosomes 7, 8, and 20, indicating different processes between APL and early-stage CRC. Mutations reinforce subtype-level differences, showing specific alterations in each subtype. **Conclusions**: These findings are especially important for developing early detection or cancer prevention tests trying to capture adenoma signatures.

## Simple Summary

Colorectal cancer originates from benign growths in the colon known as adenomas. Understanding how these adenomas become malignant is crucial to the early detection and prevention of colorectal cancer. This study examines DNA methylation and genetic differences among various types of advanced adenomas to identify potential markers that indicate progression toward cancer. By analyzing DNA methylation patterns, gene copy-number changes, and mutations in these lesions, particular signals and pathways associated with each subtype were found. These discoveries can help refine screening methods to detect distinct adenomas lesions at an early stage. By providing new insights into the initial changes leading to colorectal cancer, these findings may significantly impact approaches to prevention and early intervention strategies.

## 1. Introduction

Colorectal cancer (CRC) is a heterogeneous malignancy originating from the epithelial cells lining the colon and rectum, posing a significant global health burden with over 1.9 million new cases and 935,000 deaths in 2020 [1]. It is the third most diagnosed cancer and the second leading cause of cancer-related mortality worldwide. Despite advancements in screening and treatment, CRC continues to exhibit high mortality rates, emphasizing the need for deeper molecular insights to enhance early detection and develop targeted therapeutic interventions [2].

CRC arises through a complex interplay of genetic and environmental factors. While hereditary syndromes such as familial adenomatous polyposis (FAP) and Lynch syndrome contribute to a subset of cases [3], most CRCs occur sporadically and are influenced by lifestyle factors like diet, physical inactivity, and smoking. However, in many instances, the exact causes remain unknown, highlighting the need for further research into additional contributing factors [4,5]. The adenoma–carcinoma sequence, first proposed by Vogelstein, outlines the stepwise progression of CRC, with genetic alterations in key oncogenes and tumor suppressors such as APC [6], KRAS [7], and TP53 [8], playing pivotal roles in tumor initiation and progression. However, growing evidence underscores the role of epigenetic modifications, particularly DNA methylation changes, in CRC pathogenesis [9,10].

Aberrant DNA methylation has been identified as a major driver of CRC development by silencing tumor suppressor genes and activating oncogenic pathways [11]. These aberrant methylated regions are now recognized as potential diagnostic and prognostic biomarkers [12], offering insights into disease heterogeneity and treatment response [13]. Recent studies have revealed that CRC tumors harbor hundreds of hypermethylated regions compared with adjacent normal mucosa, highlighting the importance of epigenetic dysregulation in colorectal carcinogenesis [14,15,16].

Advanced precancerous lesions (APLs), including tubular adenomas (TAs), villous/tubulovillous adenomas (VAs/TVAs), and serrated adenomas (SAs), exhibit distinct genetic and epigenetic profiles that contribute to their variable risk of malignant transformation [17]. Serrated adenomas, whose progression is an exception to the adenoma–carcinoma sequence [18], are frequently associated with the CpG island methylator phenotype (CIMP) and microsatellite instability (MSI), further underscoring the importance of epigenetic alterations in CRC progression [19].

Although histopathology remains a cornerstone in diagnosing and subtyping APLs, accurately characterizing these lesions can be challenging. Studies report variability in assessments among pathologists, resulting in inconsistencies that may influence patient management and outcomes [20]. In certain cases, such interpretive differences can lead to uncertainties regarding lesion margins, potentially hindering timely decision making [21]. Given the complexity and heterogeneity of APLs, supplementing histopathological evaluation with additional molecular or multi-omics approaches can help enhance diagnostic accuracy and improve patient care.

Despite these challenges, no diagnostic tools have been specifically developed or validated for APLs, further underscoring the critical need for improved methodologies to ensure accurate diagnosis and effective clinical decision making. Currently, most available tools are designed for broader CRC applications, lacking specificity for APLs. A multi-omics approach, which integrates multiple layers of biological information, is essential to achieving a comprehensive and systemic understanding of the molecular landscape in CRC [22]. Such integrative analyses not only improve diagnostic accuracy but also facilitate the identification of novel, more reliable biomarkers and therapeutic targets, enabling personalized and more effective patient management [23]. Recent multi-omics studies have already provided critical insights into global DNA methylation patterns [24], gene expression, CNVs, and mutation analysis [25], as well as metabolomics [26], highlighting the potential of these advanced approaches to revolutionize the diagnosis and treatment of CRC. However, the application of these multi-omics strategies remains largely unexplored in the context of APLs, further emphasizing the need for dedicated research efforts to develop APL-specific diagnostic tools.

This study aims to identify key molecular features encompassing DMRs, CNVs, and genomic mutations across subtypes of APLs by using a multi-omics approach that integrates genome-wide data. By exploring these layers of biological information, we aim to refine APL classification and potentially improve current risk stratification models. Our findings may contribute to more informed decision making, earlier detection, and better-targeted therapeutic strategies in the management of colorectal cancer.

## 2. Materials and Methods

### 2.1. Samples

In this multi-center retrospective study, a total of 168 fresh frozen (FF) tissue samples were obtained from different international biobank repositories, including TCBN (Caen, France), VCB (Melbourne, VIC, Australia), Indivumed (Hamburg, Germany), IDIBAPS (Barcelona, Spain), LBIH Biobank (Liverpool, UK), and Dx Biosamples (San Diego, CA, USA).

A total of 96 and 46 paired tumor tissues and normal adjacent tissues from 48 APLs and 23 stage I CRCs were designedly selected for omics detection. An independent set of APL samples were used for validation purposes.

Tissue samples with different histological backgrounds (sessile serrated lesions (SSLs), tubular adenomas (TAs), or villous/tubulovillous adenomas (VAs/TVAs)) were used to obtain DNA for whole-genome bisulfite sequencing.

For our analysis, we define two samples as paired when extracted from the same patient though of differing tissue or sample type.

Demographic sample donor features are listed in Table 1 and Supplementary Table S1.

### 2.2. Sample Preparation and Whole-Genome Bisulfite Sequencing (WGBS)

Genomic DNA (gDNA) from FF tissue was extracted by using a DNeasy Blood & Tissue kit (Qiagen, Valencia, CA, USA) according to the protocol by the manufacturer. Extracted gDNA was then fragmented into segments of about 400 bp with a Covaris S220 ultrasonicator. The extracted and shredded gDNA was bisulfite-converted with the EZ DNA Methylation-Lightning kit (Zymo Research, Inc., Irvine, CA, USA). Sequencing libraries were prepared from the bisulfite-converted DNA by using the Accel-NGS Methyl-seq DNA library kit (Swift Biosciences (Ann Arbor, MI, USA) and consequently sequenced to an average depth of 22.5x with NovaSeq6000 (Illumina, San Diego, CA, USA) equipment, using paired-end sequencing (2 × 150 bp). On average, samples had about 325 million reads each.

### 2.3. Genomic Data and Methylation Processing

Quality checks and trimming were performed by using *FastQC* [27] *TrimGalore* v0.4.5 (a wrapper tool around *Cutadapt* [28], which removed adapter sequences and poor-quality bases and reads).

The remaining high-quality reads (average Phred score > 35) were aligned to a bisulfite-converted human genome (Ensembl 91 assembly, hg38) by using the *Bismark* Bisulfite Read Mapper (v0.20.0) [29], which makes use of *Bowtie* 1.2.1.1 alignment software [30]. On average, 89% of the sequences were aligned. Information regarding sequencing and alignment data quality at the sample level is reported in Supplementary Table S2.

Methylation calls for every single C analyzed were performed by the *Bismark* bismark_methylation_extractor script. For each CpG, the beta values (β) were calculated as

β = CGmethylated/(CGmethylated + CGunmethylated),

where CGmethylated is the number of methylated cytosine and (CGmethylated + CGunmethylated) is the sum of methylated and unmethylated cytosine (total number of reads) at that position. The bisulfite conversion rate was estimated from cytosine (C) in non-CpG context and in average was above 99% (98.8–99.7%).

CpGs were first selected based on their coverage: CpGs not covered by at least 5 uniquely mapped reads in all samples were discarded for subsequent analyses, such as those CpG with high beta difference variance, ending with 14,848,915 CpG sites.

### 2.4. Analyses of Differentially Methylated Position/Region

To identify differentially methylated regions (DMRs), we conducted a pairwise analysis using custom R scripts, with beta values as the input for all analyses.

Shortly, for each filtered CpG site from the previous step, a paired Wilcoxon test was applied to compare methylation levels. *p*-Values were adjusted for multiple testing by using the Benjamini–Hochberg (BH) algorithm, and CpGs with a corrected *p*-value less than 0.05 and a methylation difference of at least 0.2 between the two groups were retained. CpGs located at SNP positions with a population global minor allele frequency (MAF) greater than 1% (dbSNP Build 155) were excluded to minimize the impact of genetic variation on methylation analysis.

DMRs were defined by aggregating CpGs located within a maximum distance of 50 base pairs. Only regions containing three or more CpGs, with at least 60% of the CpGs in the region meeting the significance criteria, were selected for further analysis. This approach ensured the identification of robust regions with biologically meaningful methylation differences.

### 2.5. Pathway Enrichment Analysis

To analyze the biological meaning of our DMRs and add an extra layer of validation, we performed pathway enrichment with KEGG [31] pathways. As a first step, an extensive mapping of the DMRs was performed to find putative cis-regulatory regions related to specific genes by using the *rGREAT* function [32]. Afterwards, with the associated gene information, we proceeded to perform pathway enrichment by using *ClusterProfiler* [33], with a *p*-value of 0.05 and limiting the search to Homo sapiens. The resulting pathways were visualized by using the R package *enrichplot* [34].

### 2.6. Copy-Number Alteration Detection

To evaluate large-scale copy-number alterations (CNAs), the tool *ichorCNA* [35] was used. Read counts for each APL, CRC, and respective normal samples were calculated with the *HMMcopy* Suite [36]: each genome was divided into 10 kb non-overlapping bins, and aligned reads were counted based on overlap within each bin; then, read counts were normalized to correct for GC content and mappability biases. Centromeres were filtered based on chromosome gap coordinates obtained from UCSC (University of California at Santa Cruz) for hg38.

Normal samples were used to create a reference dataset. These data were further used to correct systematic biases arising from library construction, sequencing platform, and DNA-specific artifacts.

Copy-number analysis was performed on autosomal chromosomes only, using *ichorCNA* default parameters.

### 2.7. Somatic Mutation Detection

To overcome the bisulfite conversion problem, which can cause cytosine to appear as thymine during sequencing and complicate the detection of true mutations, a pipeline based on EpiDiverse/snp (https://github.com/EpiDiverse/snp (accessed on 28 January 2022)) [37] was created. In summary, bam files were first preprocessed by using samtools (sort, calmd, and index); then, a double-masking procedure, which manipulates specific nucleotides and base quality (BQ) scores on alignments, was applied.

For each sample pair, pileup files were created with samtools. The somatic function of *VarScan2* [38,39] was used to call somatic single-nucleotide variants (SNVs) and small insertions/deletions (INDELs) from the comparison between APLs or CRCs and NATs. Only positions that were present in both files and met the minimum coverage in both files were compared. Only mutations identified by the tool as somatic were annotated by *SnpEff* [40]. To summarize, analyze, and visualize the mutations, the VCF output from VarScan2 and SnpEff was converted into Mutation Annotation Format (MAF) files and analyzed by using the R Bioconductor package *Maftools* [41].

### 2.8. CpG Island Methylator Phenotype (CIMP)

To predict the CIMP status of each sample, we utilized CpGs derived from promoter regions that exhibited a high standard deviation (SD > 0.2) of methylation levels in tumor tissues and a low methylation level (mean β < 0.05) in normal tissues. Consensus clustering analysis based on the K-means algorithm was performed by using the R package *ConsensusClusterPlus* [42]. Given the limited number of available samples for DNA methylation profiling in this study, we classified samples with hypermethylation patterns as CIMP-H, while all other samples were categorized as CIMP-L/N.

### 2.9. Microsatellite Instability (MSI) Status Prediction

Standard short tandem repeat DNA sequence search methods are not applicable to bisulfite-converted data, as the conversion alters the DNA sequence and prevents the direct detection of microsatellite instability (MSI). To address this, we determined MSI status by examining potential frameshift mutations in genes associated with the mismatch repair (MMR) pathway, like RNF43, PMS2, MSH6, MSH2, and MLH1. Additionally, we applied the CpG biomarker panel used in the MSIMEP method [43], originally developed for microarray DNA methylation profiling, to our whole-genome bisulfite sequencing (WGBS) data. By leveraging these established biomarkers, we adapted the approach to WGBS, enabling us to estimate MSI status in our dataset effectively.

### 2.10. Principal Component Analysis (PCA)

Principal component analysis (PCA) reduces the dimensionality of multivariate data to two or three dimensions, which can be visualized graphically with minimal loss of information. PCA was performed on epigenomics data to illustrate the differences between APL histologies by using the *prcomp* function in R package stats. The R package *factoextra* was used to visualize the PCA output.

## 3. Results

**Figure 1:**
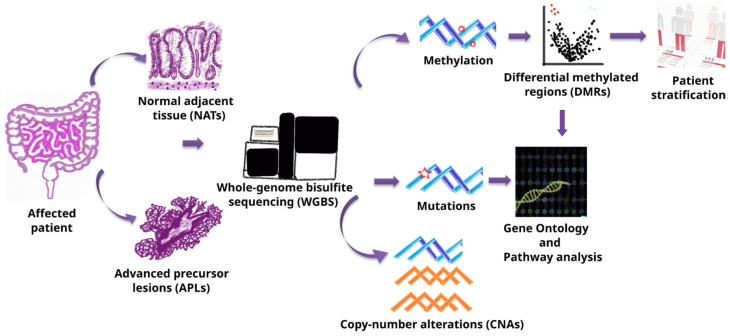
Research pipeline. Graphical representation of pipeline followed in the present work.

We obtained 168 fresh frozen samples, fully characterized clinically and histologically by using standardized criteria (described in Section 2 and Supplementary Table S1). To ensure robustness and generalizability, samples were divided into a discovery dataset of 48 APL samples paired with normal adjacent tissue (NAT), a validation dataset of 26 non-paired APL samples, and a third dataset comprising 23 paired CRC samples (Table 1). The discovery dataset was used to explore and identify the APL signature, performing differential methylation analysis and CNV/mutation analysis, while the validation dataset was used exclusively for assessing our signature performance in the agnostic identification of APLs by using the signature method. Finally, the CRC dataset was used to compare and understand the differences between the precancerous state and the invasive cancer state. The research pipeline is surmised in Figure 1.

### 3.1. DMR Analysis Shows Unique Methylation Patterns Between APL Subtypes and CRC

We conducted a comprehensive analysis to identify differentially methylated regions (DMRs) among APL subtypes. Our analysis employed two key approaches: first, pairwise comparisons of each APL subtype—SSL, TA, and VA/TVA—against their corresponding non-adjacent normal tissue (NAT) samples, allowing us to identify subtype-specific methylation changes relative to their normal tissue baseline. Second, direct comparisons among the three APL subtypes were performed to uncover DMRs that highlight methylation differences unique to each histological group.

The results of these analyses are summarized in Table 2, which presents the total number of DMRs identified in each comparison. These findings quantify the significant methylation differences observed both within individual subtypes (relative to NAT) and among the subtypes themselves. In total, 122,348 DMRs were identified, including 6263 hypermethylated and 116,050 hypomethylated regions.

**Figure 2:**
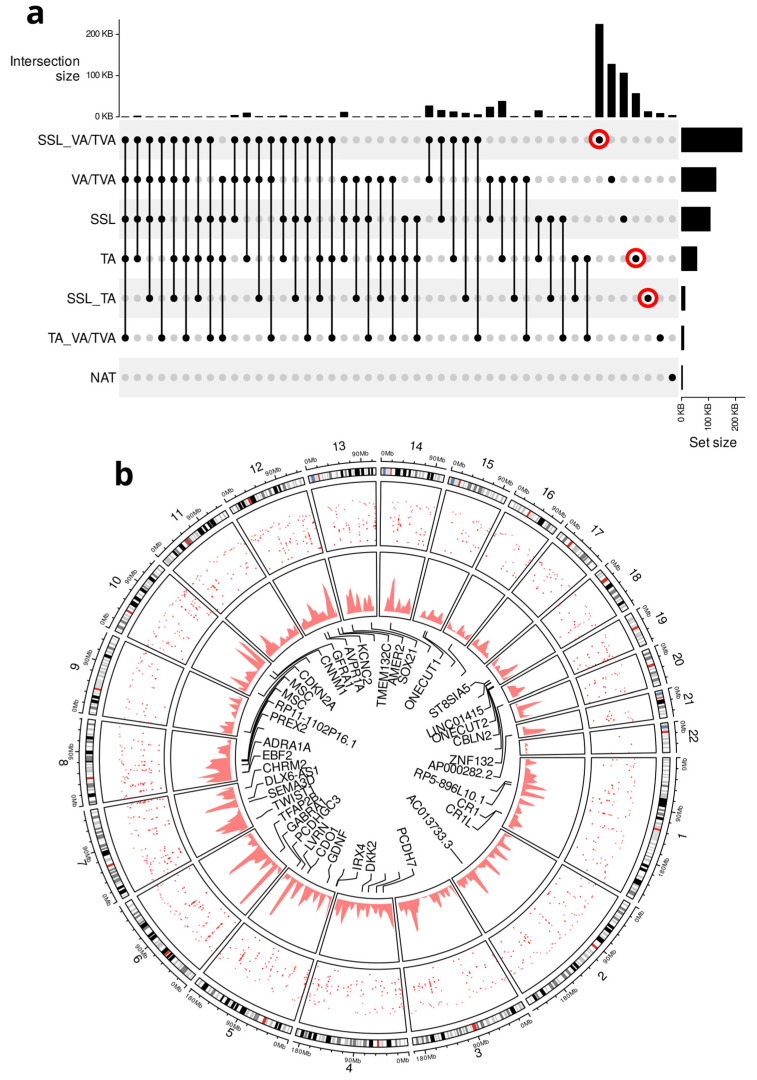
(**a**) DMR selection. The UpSet plot used to visualize the analytical comparisons among the APLs. To enrich the histological subtype signals, only those regions that do not overlap with APL vs. NAT results are considered (red circles). All identified regions and their possible intersections are represented by a black dot. (**b**) Circos plot of genomic regions, displaying the distribution of DMRs across all chromosomes. The outermost layer represents the chromosomes, followed by a dot plot indicating the genomic position of each individual DMR. The next layer is a density plot summarizing the quantity of DMRs along each chromosome. The innermost layer highlights the top 30 DMRs (annotated with gene names when applicable) that significantly contribute to the PCA analysis shown in Figure 3a.

To prioritize the most biologically relevant signals, we focused on hypermethylated DMRs unique to each APL subtype. These regions were selected based on their absence in NAT samples and their distinct presence in a single subtype, ensuring the identification of robust, subtype-specific signals. Figure 2a illustrates the different analytical comparisons performed among the three APL subtypes, with red circles highlighting the unique hypermethylated regions that represent key methylation patterns enriched in each histological subtype.

Overall, our findings reveal extensive DMRs distinguishing APL subtypes from one another, as well as from normal mucosa and CRC.

We found 2003 hypermethylated regions across the APL subtypes which were absent in the normal adjacent tissues. It should be noted that the DMRs were found not to be correlated with age (average Spearman correlation of 0.06) or sample origin (average Spearman correlation of 0.09).

After analyzing the genomic distribution of DMRs, we observed their widespread presence across all chromosomes (Figure 2b). The Circos plot provides a visual representation of the human genome, with the outermost ring corresponding to chromosomes 1 to 22. The next two rings depict the density of DMRs along the chromosome sequences, shown as a dot plot and a density plot. The innermost layer highlights the 30 most significant DMRs identified for subsequent PCA analysis, with each line representing a specific region. Gene names are included when a DMR overlaps with a known gene.

In total, 803 genes associated with DMRs were identified (Supplementary Table S3). Among these, some genes, such as *CDKN2A* and *AMER2*, are known to be epigenetically silenced in CRC. However, further transcriptomic analysis is needed to confirm their functional silencing, which is beyond the scope of the current study.

To further investigate whether specific chromosomes exhibit a higher density of DMRs, we performed an enrichment analysis of DMRs per chromosome, normalizing by chromosome length. This analysis revealed that only chromosome 7 displayed an enrichment value greater than 1.5.

By analyzing the methylation levels of the 2003 regions identified as unique to APL subtype comparisons in CRC stage I samples, we observed that CRCs exhibit distinct methylation patterns that differ markedly from those found in both APL subtypes and NATs (Figure S1). Overall, these findings suggest that the molecular origin and evolution of individual APL subtypes are associated with distinct methylation patterns that characterize their epigenetic landscapes and may play a role in the transition to colorectal cancer.

### 3.2. DMR Signature Enables Sample Stratification According to APL Subtype

**Figure 3:**
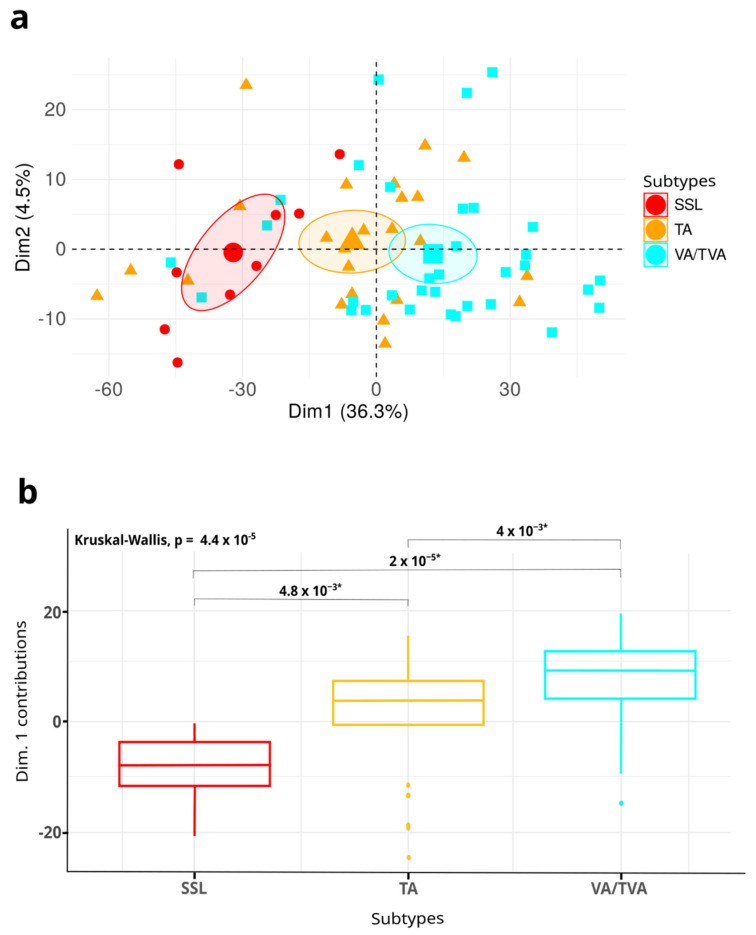
(**a**) Principal component analysis (PCA) of differentially methylated regions, showing the first two components of variance in beta values for SSL (red), TA (orange), and VA/TVA (light blue). Small symbols represent individual sample variability, while larger symbols indicate the centroid (mean) of each group in PCA space. (**b**) Boxplot illustrating the statistical differences in the first principal component’s contributions among the APL subtypes, with significance determined by the Kruskal–Wallis test.

To identify features specific to each APL subtype and reduce the influence of variables unrelated to the phenotype, we performed principal component analysis (PCA), incorporating APL samples from the validation dataset. The analysis showed that DMRs effectively segregated APL samples into three distinct groups based on the histological subtype in both the discovery and validation datasets (Figure 3a).

To statistically confirm the ability of the signature to distinguish among precancerous lesion subtypes, we conducted a Kruskal–Wallis test on the contributions of the first principal component across the three subtypes (Figure 3b). The test revealed significant differences among APL subtypes (*p* = 4.4 × 10⁻^5^). The largest difference in feature contributions was observed between SSLs and VAs/TVAs (*p* = 2 × 10⁻^5^), followed by TAs vs. VAs/TVAs (*p* = 0.004) and SSLs vs. TAs (*p* = 0.0048).

These results demonstrate that the identified methylation signature consistently stratifies samples by APL subtype with statistical significance.

### 3.3. CNA and Mutation Analysis Suggests Synergistic Mechanisms Complementary to Epigenetic Control in APLs

**Figure 4:**
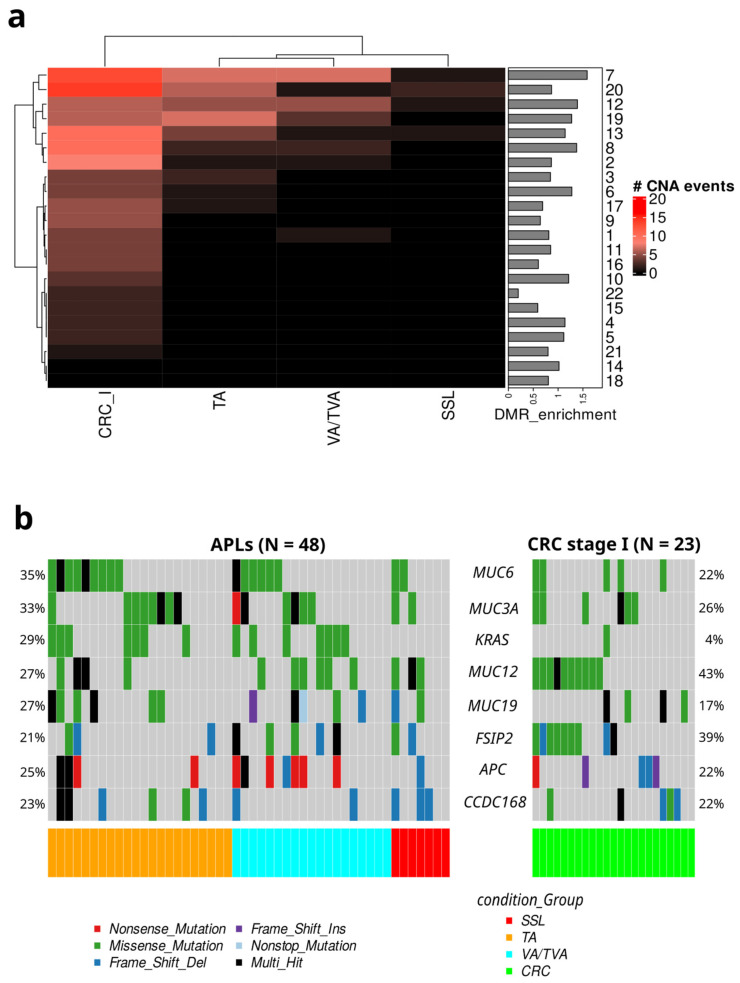
(**a**) CNA analysis. Heatmap representing the number of CNA events divided per chromosome for each APL subtype and CRC stage I. Bar plot on the right indicates the DMR regions’ enrichment per chromosome. Chr7 has a significant correlation between CNA events and hypermethylated regions. (**b**) Mafplots of frequent mutations in APLs and CRC.

Since colorectal cancer (CRC) typically develops through a multistep process involving the accumulation of both genetic and epigenetic alterations [44], we complemented our DMR analysis with an investigation of copy-number alterations (CNAs). As expected, CRC exhibited the highest number of CNA events, followed by TA and VA/TVA samples, while SSLs displayed minimal alterations (Figure 4a).

In the TA samples, the most frequent CNA events were observed in chromosomes 7, 20, 12, 19, 13, 8, and 3, with fewer alterations detected in chromosomes 6 and 17. Similarly, the VA/TVA samples showed CNA events primarily in chromosomes 7, 12, and 19, with occasional events in chromosomes 8 and 1. In contrast, SSLs exhibited CNAs predominantly in chromosomes 20, 12, and 13, suggesting distinct underlying mechanisms driving copy-number alterations in each APL subtype. These findings align with the histopathological differences among APLs, further highlighting the unique molecular characteristics of SSLs compared with VAs/TVAs and TAs.

The widespread CNA events detected in CRC samples are consistent with previously described chromosomal aberrations in colorectal tumors, reinforcing the notion that genetic alterations contribute to CRC progression alongside epigenetic modifications.

Since APC and KRAS are the most frequently mutated driver genes in the adenoma-to-carcinoma sequence [6], they could be expected to demonstrate alterations more frequently; moreover, the MUC6 and MUC3A mutations were the most frequent, followed by KRAS mutations, principally in TAs and VAs/TVAs (Figure 4b). However, most SSL samples exhibited alterations in neither KRAS nor APC.

The integration of CNA and mutation analysis with differential methylation analysis provides a comprehensive view of the molecular landscape of APLs. Our analysis suggests that there is a subtle distinction between genetic events in the different histological subtypes of APLs: TAs and VAs/TVAs present a stronger signal in CNA analysis, while SSL presents the weakest signal in CNAs, as well as almost no hits in KRAS or APC. Early-stage CRCs exhibit a different mutation profile compared with APLs, with notable alterations in MUC12 and FSIP2, observed in 43% and 39% of cases, respectively, with a single KRAS occurrence and 22% of APC mutations detected. These findings suggest that both CNAs and mutations in specific genomic regions contribute to the differences observed between the APL subtypes and CRC.

### 3.4. Pathway Enrichment Analysis Reveals Redundancy in Circuitry of Transcriptional Regulation Cellular Fate and Proliferation Signaling in APL Progression

**Figure 5:**
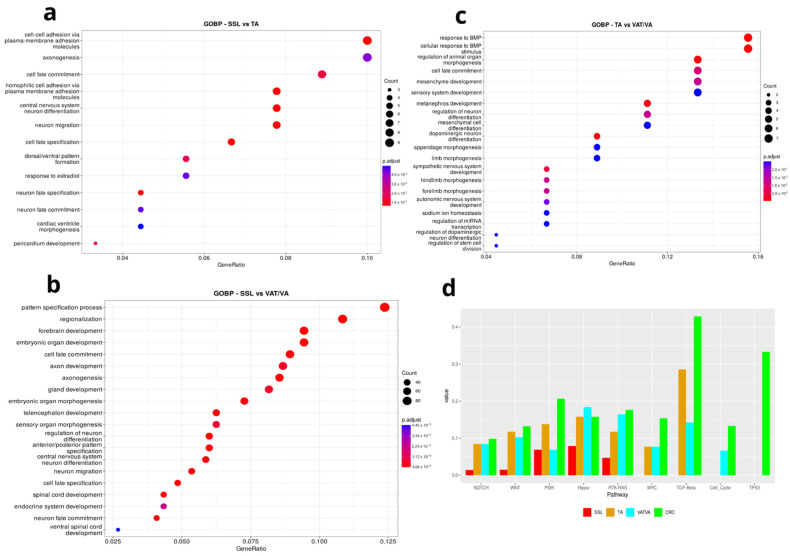
(**a**–**c**) **Gene Ontology (GO) enrichment analysis of differentially methylated regions (DMRs) identified in various APL comparisons**. Dot plot showing the GO terms for the identified differentially methylated genes. The X-axis represents the gene ratio, and the Y-axis is the functional category. Dots are color-coded from blue to red based on the adjusted *p*-value. The size of the dots is proportional to the gene count. (**a**) GO enrichment in biological processes in SSL vs TA comparison. (**b**) GO enrichment at biological processes in SSL vs. VAT/VA comparison. (**c**) GO enrichment in biological processes in TA vs VAT/VA comparison. (**d**) Oncological pathway enrichment. The enrichment of mutated genes in known oncogenic pathways indicates differences among subtypes but also between APL and CRC stage I.

To investigate the biology underlying the differential methylation signal identified, we performed a Gene Ontology (GOs) and pathway enrichment analysis based on DMRs obtained from the APLs that revealed distinctive subtype dysregulation in multiple biological pathways (Figure 5).

In the SSL vs. TA comparison (Figure 5a), we found several morphogenic and cell fate processes, such as cell–cell adhesion, cell fate commitment and specification, and dorsal/ventral pattern formation. Moreover, when comparing SSLs vs. VAs/TVAs (Figure 5b), we found slightly different processes but still related to cell fate, such as the pattern specification process, regionalization cell fate commitment, gland development, plasma membrane adhesion molecules, anterior/posterior pattern specification, and cell fate specification.

Finally, a TA vs. VA/TVA comparison (Figure 5c), showed enrichment in processes such as cell fate commitment, the regulation of epithelial cell proliferation, miRNA transcription, and the regulation of stem cell proliferation, among other terms related to tissue reorganization.

These results suggested that genes affected by the DMRs in each subtype showed unique traits but also redundancy at the pathway level, which support cellular proliferation, morphogenesis, and tissue reorganization. These molecular events suggest that cellular reorganization and the potential to reorganize tissue structures are critical to the development of APLs, which is consistent with the known clinical evolution of APLs and CRC.

Oncological pathway enrichment analysis using the somatic mutations found in APLs and early-stage CRC (Figure 5d) elucidates interconnected signaling pathways that help to alter the normal colonic structure and, in the case of CRC, contribute to an invasive phenotype. Crosstalk among dysregulated pathways, such as NOTCH, WNT, Hippo, and RTK-KRAS, underscore the complexity of molecular events driving lesion progression. Interestingly, the PI3K, MYC, TGF-Beta, cell cycle, and TP53 pathways are more affected in CRC than in APLs. It is important to note that there are substantial differences among subtypes: SSLs exhibit a lower proportion of their pathway-associated genes altered; TAs and VAs/TVAs show a higher enrichment in TGF-beta signaling but similar to normal tissue in other pathways; and VA/TVA is the only subtype that shows enrichment in the cell cycle pathway.

Collectively, these results provide a comprehensive molecular portrait of APLs, highlighting the convergence of transcriptional misregulation and oncogenic signaling in their progression from normal tissue to APLs and then to CRC.

### 3.5. DMR Phenotype in APLs Is Not CIMP- and MSI-Related

To investigate whether the phenotype associated with DMRs is related to the CpG island methylator phenotype (CIMP) status, we analyzed specific methylation patterns in promoter regions. Regions exhibiting hypermethylation were classified as CIMP-H, while those without clear hypermethylation or hypomethylation were categorized as CIMP-L/N (Supplementary Figure S2a). Unexpectedly, the DMR-driven phenotype appeared independently of the classical CIMP classification. This lack of strong correlation suggests the potential involvement of alternative mechanisms driving differential methylation in advanced precancerous lesions.

The microsatellite instability (MSI) status was assessed by using the MSIMEP method (Supplementary Figure S2b). Our analysis showed no significant association between the MSI status and the identified DMR phenotype. The observed epigenetic alterations in APLs appear to be distinct from the genomic instability typically associated with MSI.

These findings indicate that the phenotypical differences observed in APLs may not be directly linked to the CIMP status or MSI. The absence of strong correlations with these markers suggests that alternative molecular mechanisms likely contribute to the distinct methylation patterns and phenotypic differences in advanced precancerous lesions.

## 4. Discussion

Here, we present evidence of aberrantly methylated regions, supported by chromosomal alteration and somatic mutations, in advanced precancerous lesions.

Since identifying and analyzing methylation pattern differences among APL subtypes provides valuable insights into the epigenetic alterations that precede CRC, this study focused on the significant DMRs detected across various APL subtypes, highlighting their potential roles in the progression from normal colonic mucosa to malignancy. Our findings suggest that these epigenetic changes are subtype-specific and may contribute to the heterogeneity observed in CRC.

The hypermethylation of regions related to genes involved in cell cycle regulation, DNA repair, and apoptosis observed in our study suggests that these pathways are crucially disrupted in the early stages of colorectal pre-neoplasia, in agreement with previous knowledge. For example, the methylation of the CDKN2A gene, a well-documented event in colorectal adenomas [45], was confirmed in our dataset. This methylation is known to happen early and to increase through the adenoma-to-carcinoma progression. Other genes, such IRX4, are known to be silenced epigenetically and under-expressed in pancreatic cancer [46] or AMER2 (also called FAM123A), which is known to be a negative regulator of WNT signaling and its aberrant methylation and to be correlated to progression in precancerous stages in gastric carcinomas [44].

Our study identified distinct DMR signatures that enable the stratification of samples according to their APL subtype. The subsequent unsupervised clustering of these DMRs demonstrated a clear separation of samples into their respective subtypes, indicating the robustness of these methylation markers. Our findings were validated by using an independent cohort. These DMR signatures not only facilitate the precise stratification of APL samples but also hold potential as biomarkers for early detection and personalized treatment strategies in clinical settings.

The analysis of CNVs revealed notable differences among the premalignant lesion subtypes. CNVs are known to contribute to genomic instability, a hallmark of cancer [47], and their presence in premalignant lesions underscores their role in early carcinogenesis [48]. SSLs exhibited fewer genomic gains compared with VAs/TVAs, with gains primarily concentrated on chromosomes 7, 12, 13, and 20. Additionally, SSLs displayed the lowest frequency of genomic losses compared with other APL subtypes, highlighting distinct patterns of chromosomal alterations across these lesions.

Here, it is important to highlight that chromosome 7 is enriched in CNVs for TAs and VAs/TVAs as well as for DMRs (Figure 2b), and a possible transformation pathway has been reported before in CRC and adenomas [49]. Interestingly, all the Mucin genes included in our mutation analysis are encoded on chromosome 7. This family of glycoproteins has been implicated in chronic inflammatory states and the promotion of oncogenesis in numerous malignancies, especially CRC [50].

These findings can inform the development of subtype-specific diagnostic and therapeutic strategies. Further research is needed to understand the functional impact of these CNVs and their potential as biomarkers for early detection.

Somatic mutations are pivotal to the initiation and progression of CRC and are known to be linked to epigenetic modifications [51]. Our study identified several recurrent mutations across different premalignant lesion subtypes, providing insights into the molecular events that may drive their progression to invasive malignancies.

KRAS, TP53, and APC mutations were predominantly found in VAs/TVAs, while SSLs showed mutations in APC and TP53. This aligns with known molecular pathways involved in the adenoma-to-carcinoma sequence in VAs/TVAs but represents a finding previously unreported in the serrated pathway.

SSLs exhibited a higher mutational burden compared with the VA/TVA samples, indicating more heterogeneous clonal evolution. This suggests that SSLs may have a greater potential for malignant transformation due to their diverse mutational landscape. Our findings also indicate that the identified signature in the SSL subtype is not associated with CIMP or MSI events. The independence of this epigenetic signature suggests that multiple pathways drive methylation changes during adenoma progression, as reported by other studies [52]. This highlights the need to explore distinct molecular mechanisms in precancerous lesion progression. However, further studies are necessary to investigate this topic in detail.

Identifying these somatic mutations provides valuable insights into the molecular heterogeneity of premalignant lesions. Large-scale studies have aimed to identify molecular signatures through mutations [53], differential methylation [54,55,56], gene expression [57], proteomics [58], fragmentomics [59], and glycoproteome analysis [60]. By integrating epigenetic signatures with genomic alterations, our study offers a complementary perspective on early CRC development. It underscores the importance of methylation-based stratification of APLs and its potential application in plasma-based profiling.

The analysis of biological pathways has uncovered notable redundancy in the transcriptional regulation circuitry that governs cellular fate and proliferation signaling in APLs. This redundancy, manifested through multiple overlapping pathways, ensures robust control of critical cellular processes despite genetic and epigenetic alterations. Such redundancy may facilitate malignant transformation by providing alternative routes for sustaining proliferative and survival signals. These findings underscore the complexity of transcriptional networks in CRC progression [61] and highlight potential challenges and opportunities for therapeutic interventions targeting these redundant pathways to effectively disrupt the progression from premalignant lesions to invasive carcinoma. Further, understanding these differences could aid in developing targeted prevention strategies and improve the precision of early detection methods.

We are aware that our study has limitations that should be acknowledged. One key challenge lies in mutation calling from WGBS data, as the bisulfite treatment process can introduce artifacts that mimic genuine mutations, potentially leading to false-positive calls. To mitigate this, we implemented a robust pipeline with a double-masking procedure and stringent filtering criteria, which effectively reduces false positives but may increase the risk of false negatives, particularly in regions with low sequencing coverage.

Additionally, the validation of our identified biomarkers requires larger, independent cohorts analyzed under double-blinded conditions to minimize bias and enhance clinical relevance. Further functional studies are also needed to clarify the biological interplay between methylation changes and chromosomal aberrations in colorectal cancer progression.

Moreover, our approach to defining a “CIMP-like” phenotype, based on promoter-region CpGs with high variability in tumors and low methylation in normal tissues, diverges from traditional CIMP classification methods, such as those using predefined marker panels or clustering-based approaches. While this non-standard strategy enhances our ability to capture biologically relevant epigenetic alterations beyond classical definitions, we acknowledge that differences in methodology may contribute to observed variations. Nevertheless, the comprehensive genome-wide coverage provided by WGBS, which assays a significantly larger portion of the genome compared with methylation arrays, strengthens our capacity to identify novel hypermethylation signatures that could provide complementary insights into colorectal cancer progression.

Despite these limitations, our findings offer a valuable perspective on epigenetic heterogeneity and emphasize the importance of continued research to validate and refine our approaches across diverse populations.

## 5. Conclusions

Our integrated analysis of differentially methylated regions (DMRs), copy-number variations (CNVs), and somatic mutations across various subtypes of advanced premalignant lesions (APLs) of the colon provides new insights into the molecular alterations driving early colorectal carcinogenesis. By combining epigenetic, genomic, and mutational data, we identified distinct molecular signatures associated with each APL subtype, highlighting the heterogeneity within these lesions and their unique progression pathways toward malignancy.

The findings underscore the potential of DMRs as robust biomarkers for early detection, offering opportunities to improve diagnostic accuracy and stratify patients based on their risk of progression to colorectal cancer (CRC). Additionally, the observed differences in CNVs and somatic mutations among APL subtypes further reinforce the importance of a multi-omics approach to fully characterize the molecular landscape of these lesions.

Beyond their diagnostic value, these molecular alterations present opportunities for the development of targeted preventive strategies. Epigenetic modifications offer an attractive avenue for therapeutic intervention due to their reversible nature. The identification of subtype-specific alterations could pave the way for personalized medicine approaches, enabling tailored surveillance and treatment strategies for individuals at higher risk of CRC progression.

While our study provides a comprehensive framework for understanding the molecular biology of APLs, further research is required to validate these findings in larger cohorts and to explore their functional relevance. Future studies should also investigate the integration of these molecular signatures into existing clinical workflows to enhance early detection and improve patient outcomes. Finally, the incorporation of artificial intelligence and machine learning algorithms must be performed to improve feature selection and detection rates.

In conclusion, this study contributes to the growing body of knowledge on early colorectal tumorigenesis and highlights the potential of multi-omics strategies to transform the diagnosis, prevention, and management of CRC. By advancing our understanding of APL subtypes, we hope to facilitate the development of novel tools and strategies aimed at reducing the global burden of colorectal cancer.

## Figures and Tables

**Table 1:** Relevant characteristics of patients in this study.

	APL	APL (Validation)	CRC Stage I
**n**	**48**	**26**	**23**
**Age median (IQR), years** **(range)**	**68.5 (14.75)** **(39–86)**	**68.5 (19.75)** **(23–84)**	**68 (13)** **(37–81)**
**Gender female/male, n**	**17/31**	**16/10**	**14/9**
**Histology**			
**SSL, n**	**7**	**7**	
**TA, n**	**22**	**4**	
**VA/TVA, n**	**19**	**15**	

**Table 2:** Summary of DMRs in all analyses.

Comparison	DMRs	Hyper DMRs	Hypo DMRs
**SSL vs. SSL NAT**	**6317**	**1271**	**5046**
**TA vs. TA NAT**	**36,297**	**861**	**35,436**
**VA/TVA vs. VA/TVA NAT**	**65,794**	**1807**	**63,987**
**SSL vs. TA**	**1299**	**137**	**1162**
**SSL vs. VA/TVA**	**11,955**	**2096**	**9859**
**TA vs. VA/TVA**	**651**	**91**	**560**
**SSL NAT vs. TA NAT**	**4**		
**SSL NAT vs. VA/TVA NAT**	**30**		
**TA NAT vs. VA/TVA NAT**	**1**		

## Data Availability

The data presented in this study are available on a reasonable request from the corresponding author.

## Supplementary Materials

The following supporting information can be downloaded at: https://www.mdpi.com/article/10.3390/cancers17040654/s1, Figure S1: Heatmap of methylation profiles in all the groups, Figure S2: (**a**) Heatmap of CIMP status. (**b**) Heatmap of MSI status, Table S1: Description of the samples included in the study, Table S2: Data quality metrics per sample, Table S3: List of genes with at least one associated DMR, Table S4: Comprehensive list of somatic mutations identified in the study.
