# Supplementary figures and images for: Colorectal Adenoma Subtypes Exhibit Signature Molecular Profiles: Unique Insights into the Microenvironment of Advanced Precancerous Lesions for Early Detection Applications
Source: Cancers (Basel). 2025 Feb 14;17(4):654. doi: 10.3390/cancers17040654 (PMC11852906; doi:10.3390/cancers17040654)

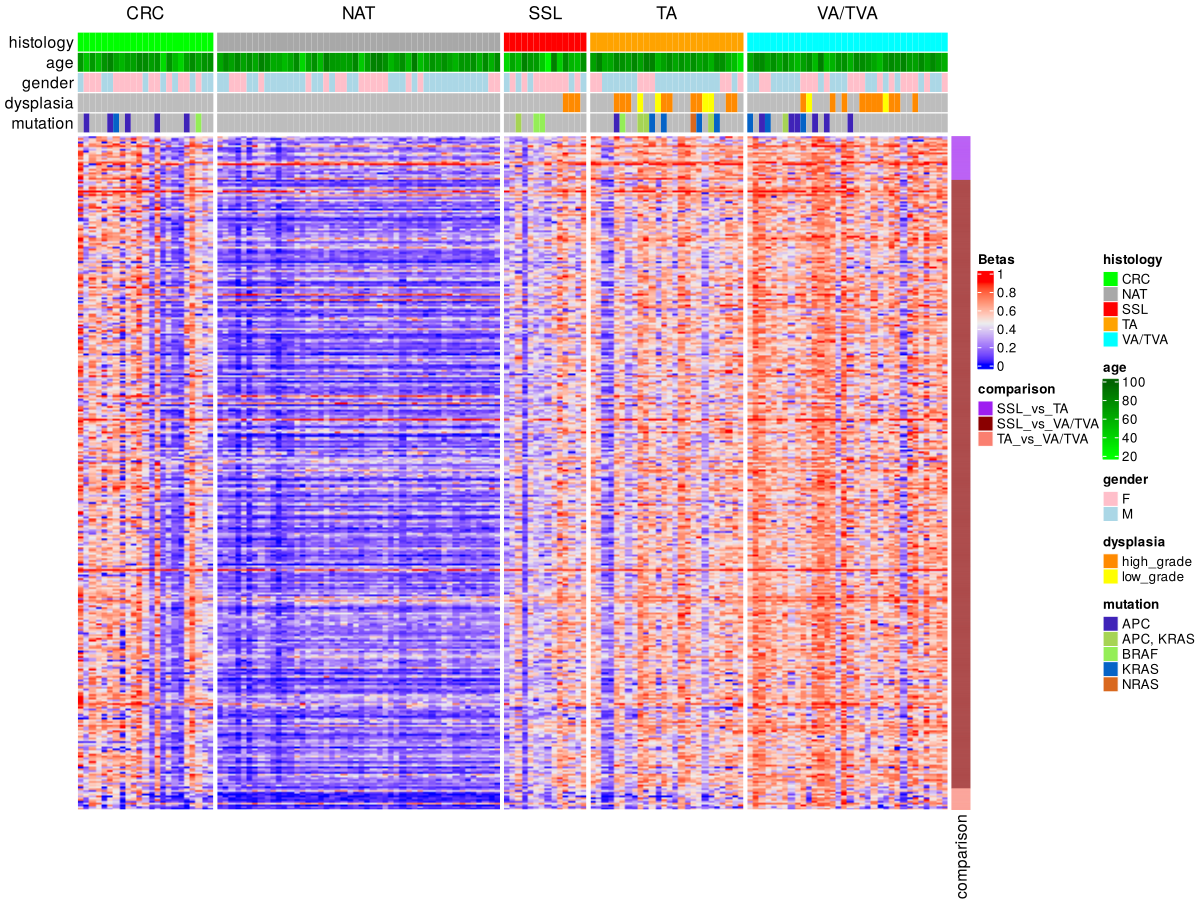

Supplement: Supplementary file 1 [file cancers-17-00654-s001.zip › FMMancuso_et_al - Supplementary_fig_S1.png]

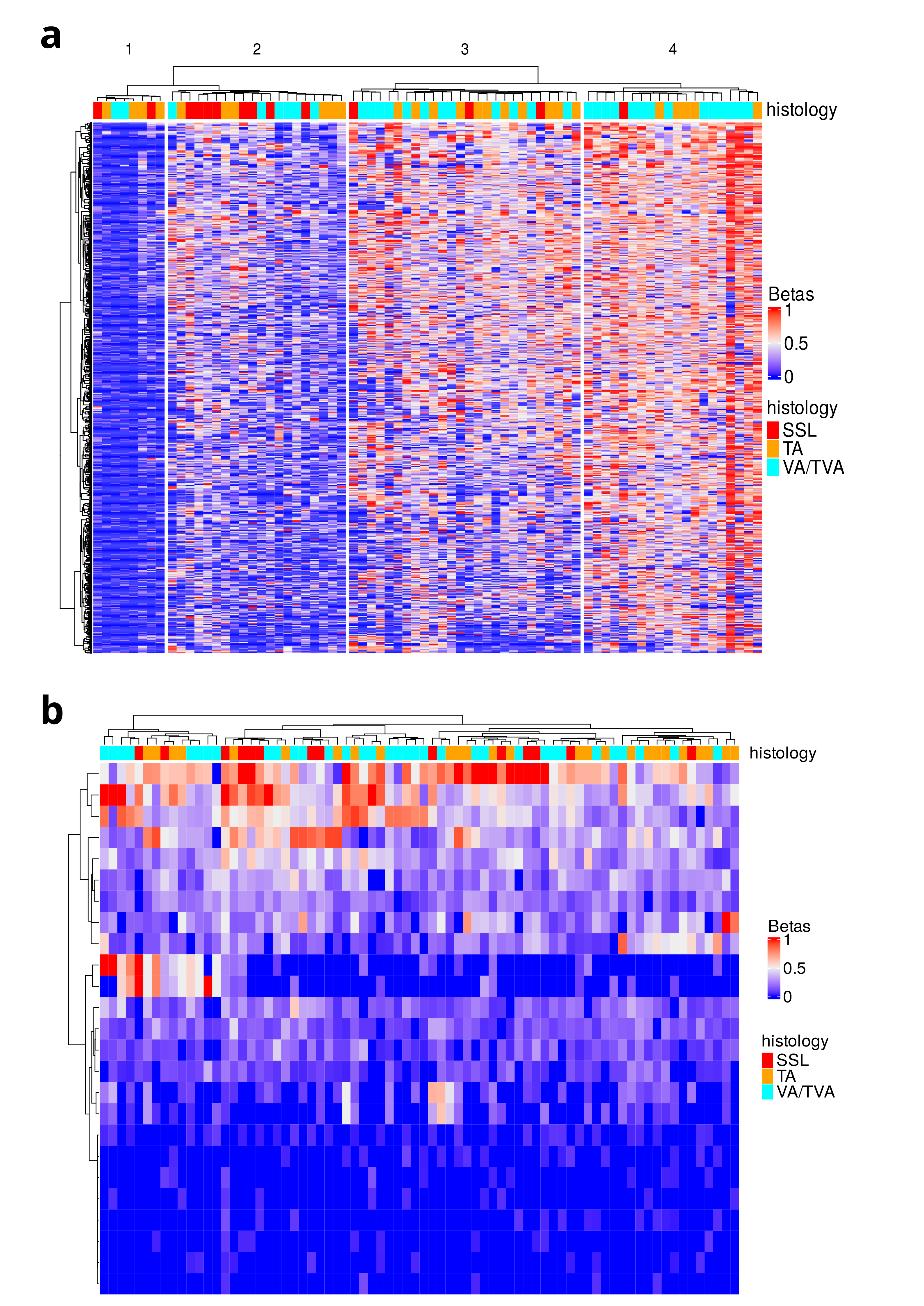

Supplement: Supplementary file 1 [file cancers-17-00654-s001.zip › FMMancuso_et_al - Supplementary_fig_S2.png]
